# Genome-wide DNA methylation analysis of hippocampal tissue in a murine model of attention deficit-hyperactivity disorder

**DOI:** 10.1371/journal.pone.0323756

**Published:** 2025-06-04

**Authors:** Rodrigo Vidal, Ignacio Lopez, Gonzalo Ugarte, Darwin Contreras, Ricardo Piña, Felipe Godoy, Carlos Rozas, David Rubio, Carola Mantellero, Luis Constandil, Jorge Escobar, Bernardo Morales

**Affiliations:** 1 Department of Biology, Faculty of Chemistry and Biology, Laboratory of Genomics, Molecular Ecology and Evolutionary Studies, University of Santiago of Chile, Santiago, Chile; 2 Department of Biology, Faculty of Chemistry and Biology, Laboratory of Neuroscience, University of Santiago of Chile, Santiago, Chile; 3 Facultad de Ciencias de la Rehabilitación y Calidad de Vida, Escuela de Kinesiología, Universidad San Sebastián, Santiago, Chile; 4 Department of Biology, Faculty of Sciences, Metropolitan University of Education Sciences, Santiago, Chile; 5 Dirección de Investigación, Universidad Bernardo O’Higgins, Santiago, Chile; 6 Faculty of Life Sciences, Andrés Bello National University, Santiago, Chile; 7 Department of Biology, Faculty of Chemistry and Biology, Laboratory of Neurobiology, University of Santiago of Chile, Santiago, Chile; 8 Facultad de Ciencias, Instituto de Química, Pontificia Universidad Católica de Valparaíso, Valparaíso, Chile; Shiga Medical Center, JAPAN

## Abstract

Attention Deficit-Hyperactivity Disorder (ADHD) is a neurodevelopmental disorder with a prevalence around 5% in children and adolescents and 2.5% in adults. Recent reports using GWAS approaches have identified several genetic risk loci for this disorder. However, the epigenetic influence of extrinsic factors during pregnancy or the exposure to environmental factors during childhood, on the onset of the disorder remains unclear. This question has been addressed by analyzing blood or saliva samples from ADHD patients or by postmortem analysis. The aim of this study was to determine differential patterns in DNA methylation in fresh hippocampal samples using a murine model of ADHD. We analyzed the genome-wide pattern of differentially methylated CpG sites using the Illumina Infinium Mouse Methylation BeadChip in fresh hippocampal samples from the prenatal nicotine exposure (PNE) mouse model of ADHD and control animals. Our analysis revealed 218 DMPs including genes associated with growth factors signaling, such as adhesion G protein-coupled receptor B2 (*ADGRB2*), leukemia inhibitory factor receptor (*LIFR*) and erb-b2 receptor tyrosine kinase 3 (*ERBB3*) involved in synaptogenesis, proliferation, and differentiation of neural stem cells. The functional gene enrichment analysis of differentially methylated positions (DMPs) revealed the nervous system development as the biological process with highest enrichment factor. In addition, the GO and KEEG enrichment analysis of 113 differentially methylated regions (DMR) revealed several loci associated with the positive regulation of Hippo signaling (involved in neuronal development) in PNE samples. In addition, our results revealed a DMP previously associated to ADHD patients supporting the PNE murine model of ADHD. These results are relevant in terms of the validation of PNE model of ADHD and for the identification of epigenetic markers of the disorder in humans. In addition, our results are relevant for the characterization of the cellular and molecular mechanisms underlying the ADHD, currently unknown.

## Introduction

Attention Deficit-Hyperactivity Disorder (ADHD) is a neurodevelopmental disorder with a high prevalence (approximately 5%) among children worldwide and is characterized by hyperactivity, inattention and/or impulsivity, which affect learning and sociability at school [1]. The prevalence of ADHD in children and adolescents has a male-female ratio of approximately 4:1 in clinical studies and in adults between 2:1 and 1:1 [2,3].

Several studies support a polygenic cause for ADHD and nongenetic factors involved in the etiology of this disorder. Extrinsic factors such as smoking and alcohol consumption during pregnancy, exposure to contaminants such as lead, diet deficiencies and low educational attainment have been correlated with ADHD [4]. Recently, a genome-wide association study (GWAS) meta-analysis, involving thousands of ADHD and control individuals, identified 27 risk loci for ADHD involving genes that are expressed mainly in excitatory and inhibitory neurons of the frontal cortex [5]. However, the mechanism of interaction among the genetic background and environmental factors involved in the ADHD onset remains unclear. In this context, DNA methylation, an epigenetic biological process that regulates gene expression, may arbitrate environmental or genetic effects. Several studies focused on epigenetic modifications associated with ADHD have been reported [6]. These reports correspond to epigenome-wide analysis studies (EWASs) using different types of samples (peripheral blood or saliva) taken from children, adolescents, or adults. Notably, few common loci with different methylation statuses between control and ADHD patients such as *VIPR2* encoding the receptor for vasoactive intestinal peptide (VIP) expressed in the CNS and regional methylation patterns of neurotransmitters-associated pathways have been identified [7–9]. Although these studies have provided important knowledge about gene methylation patterns in ADHD, the most of them have been performed on peripheral blood or saliva where medium-large scale noninvasive sampling is feasible [10–12]. The DNA methylation pattern is tissue- and cell type-specific [13,14], so it is not clear whether ADHD blood DNA methylation results may act as a proxy for the DNA methylation patterns of target ADHD tissues. In this context, investigating the primary ADHD-related tissues, such as the hippocampus, is extremely relevant for advancing our understanding of the cellular and molecular mechanisms involved in ADHD. The meta-analysis of neuroimaging data obtained from children, adolescents and adults with ADHD revealed statistically significant reductions (compared to control groups) in cortical thickness, cortical surface area and the volume of subcortical areas such as the accumbens, amygdala, caudate, putamen and hippocampus. Interestingly, this brain area which is critical for learning and memory processes, remains significantly reduced during later stages of development [15].

The access to brain samples from DHD patients has been limited to postmortem analysis [16], lead to the development and characterization of animal models of the disorder. Several rodent models of ADHD have been described including genetic models such as *SNAP-25 Knockout (KO)* mice, *DAT KO* mice and *LPHN3 KO* mice and rats, those induced by exposure to environmental chemicals such as lead and pesticides, and those induced by prenatal exposure to nicotine or alcohol. The prenatal nicotine exposure (PNE) murine model presents several symptoms described in ADHD patients such as increased locomotor activity, low performance in cognitive tests, inattention, and transmissibility until the third generation [17]. In addition, PNE mice exhibit a reduction in dopamine turnover in the frontal cortex and striatum and a significant volume reduction in the cingulate cortex [18]. Our previous works supported the correlation of hyperactive and inattention behaviors in PNE mice with impairment hippocampal long-term potentiation (LTP). These abnormalities were reversed after a single administration of methylphenidate [19]. In the present study we utilized the PNE mouse model of ADHD and the recently developed Infinium Mouse Methylation BeadChip platform (Illumina) to evaluate the genome-wide DNA gene methylation patterns in hippocampus and the pathways associated with this disorder. Our PNE animal ADHD model corroborates some methylation patterns observed in humans and reveals new differentially methylated GpC sites in the hippocampal tissue.

## Materials and methods

### Murine model of ADHD

Protocol for the care and use of animals were approved by the Bioethical Committee of the University of Santiago of Chile. Prenatal nicotine exposure (PNE) mice were obtained following the protocol described in (18). Briefly, C57BL/6 female mice were treated with 0.1 mg/ml nicotine for three weeks before mating and during the entire pregnancy period. Nicotine was administered orally and dissolved in the drinking water with 2% saccharine ad libitum. The behavioral characterization of PNE and control mice is shown in the S1 Fig.

### Tissue collection

PNE and control mice at postnatal day 21 (P21) were sacrificed by decapitation under halothane anesthesia. The brains were removed and transferred into ice-cold dissection solution containing the following (in mM): 125 NaCl, 4 KCl, 10 glucose, 1.25 NaH_2_PO_4_, 25 NaHCO_3_, 0.5 CaCl_2_, and 2.5 MgCl_2_ (pH 7.4), which was subsequently equilibrated with a 5% CO_2_–95% O_2_ mixture. Transversal 300 µm brain slices containing the hippocampal area were obtained using a vibratome (Leica, Nussloch, Germany) and the hippocampi were further dissected under a low-magnification microscope.

### DNA isolation and bisulfite conversion

Bilateral hippocampal tissues from six control (3 males and 3 females) and six treated groups (3 males and 3 females) were homogenized shortly after sacrifice and subsequently handled for DNA isolation with the DNeasy Blood and Tissue Kit (Qiagen, Hilden, Germany) following the manufacturer’s instructions. Purity, DNA concentration, and integrity were obtained with a Qubit 4 fluorometer (Thermo Fisher Scientific, MA, USA) and a 2100 Bioanalyzer System (Agilent, CA, USA), respectively. For each sample, one microgram of genomic DNA was utilized for bisulfite conversion, employing the EZ DNA Methylation Kit (Zymo Research, Irvine, CA, USA) and DNA methylation was determined with the Infinium Mouse Methylation BeadChip (Illumina, CA, USA) (GPL30650), following the manufacturer’s recommendations.

### DNA methylation analysis

#### Identification of differentially methylated positions (DMPs) and differentially methylated regions (DMRs).

IDAT files (GSE290914) were exported into R and the Champ package [20] and the GenomeStudio Methylation Module (v1.8) and utilized to perform subsequent analysis, including removal of probes with detection p > 0.01, mapping to Y and X chromosomes, and <3 beads in at least 5% of the samples. The methylation ratios of the CpG sites were described by beta-values (ranging from 0, no methylation to 1, full methylation). Then, the beta-values data were normalized with the MIxture Quantile dilation option [21], amending type I and type II probe bias. To identify general methylation patterns, a principal component analysis (PCA) on methylation levels in all samples was realized using the prcomp library in R (https://github.com/SurajGupta/r-source/blob/master/src/library/stats/R/prcomp.R). Differential methylated CpG sites were determined using a linear model with the package limma and included sex as a covariate. CpGs with a corrected (Benjamini–Hochberg) P-value <0.05 and an absolute Δbeta > 0.20 were considered as DMP. In addition, DMRs were identified with the LASSO methodology [22] and the option ChAMP.DMR, including a minimum of 3 successive CpG sites and a distance no greater than 1,000 bp among 2 contiguous DMPs. DMRs with a corrected (Benjamini–Hochberg) P-value <0.05 were considered as significant DMR. The ChAMP.import option was utilized to annotate all DMRs. The R package ClusterProfiler (version 3.14.3) was utilized to perform analysis for enriched Gene Ontology (GO) and Kyoto Encyclopedia of Genes and Genomes (KEGG) pathways of DMRs and DMPs.

#### RT-qPCR.

Three DMR genes were randomly selected for RT-qPCR analysis. Total RNA was isolated using Trizol (Invitrogen, USA) and RNeasy Mini KIT (Qiagen, USA) and the genomic DNA residue was removed with TURBO DNAse treatment (Invitrogen, USA), according to the manufacturer’s instructions. The qualities, concentrations, and integrities of RNA extracts were evaluated with a Qubit 3.0 fluorometer (Life Technologies, USA; Qubit RNA HS kit) and a Bioanalyzer 2100 (Agilent Technologies, USA; RNA Nano 6000 Assay Kit). Reverse transcription was performed with a blend of random primers and oligo(dT) and iScript reverse transcriptase (iScript cDNA synthesis kit, Bio-Rad, USA) according to the manufacturer’s instructions. DMR specific primers for each of the selected genes were designed with Primer3 Plus (https://www.primer3plus.com/index.html; *CORO7,* F:5’TACTGGGCATTGTGCCTCTG-3´, R:5´AAGGGCGAGAAGTCCAAGTC-3´; *ERBB3,* F:5’-CTTGCCTCGATGTCCTAGCC-3´, R:5’- CTGAAAAGCAAGCCCAGCAC-3´; *MYL4*, F:5´-GCTCATCTCTCTGGCTGCTC-3’, R:5´- ACAGGCTGATCCCTTCTTGC-3’). Quantitative real-time PCR was conducted using RT² SYBR Green ROX qPCR Mastermix (Qiagen, USA) and Rotor-Gene Q (Qiagen). qPCR cycling consisted of 95°C for 4 min, followed by 40 cycles of: 95°C for 6 s and 60°C for 45 s, with a final melting curve analysis at 95°C for 12 s, 60°C for 60 s, and 95°C for 15 s. All RT-qPCRs were performed in triplicate and the Rpl13a gene was utilized as normalizer [23]. The relative expression of the selected genes was estimated using the 2^–ΔΔCt^ method [24]. The PCR efficiency of each pair of primers was calculated with the LinRegPCR V. 2021.2 package, based on raw fluorescence data [25].

## Results

### Overall methylation profile of hippocampal samples from PNE and control mice

**Fig 1:**
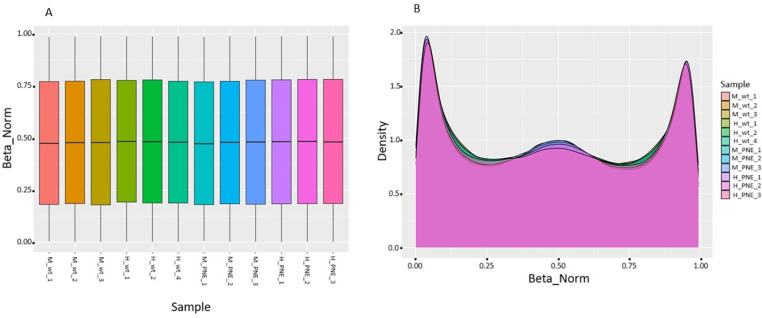
Overall CpG methylation pattern in control (wt) and PNE mice. (A) Box plots showing the methylation profiles of male (M) and female (H) hippocampal cells from control (wt) and PNE mice. (B) Density plot of the averaged β-values of male (M) and female (H) CpG sites of controls (wt) and PNE mice.

After quality control of the chips and samples, box plots were used to characterize the data. The results revealed that the β-normalized experimental values were uniformly distributed, implying that the data obtained were proportional. A density plot revealed a similar bimodal methylation pattern among control and PNE mice, with 2 peaks at higher (~0.85) and lower (~0.07) β-average values (Fig 1).

**Fig 2:**
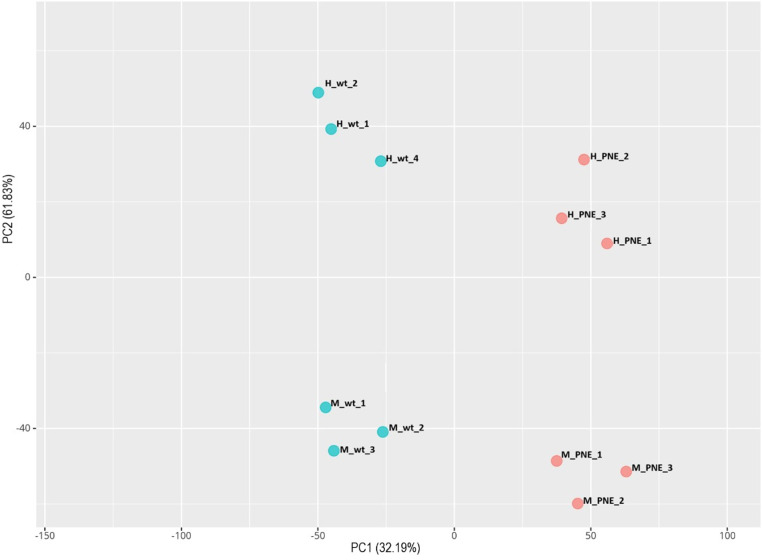
Overall DNA methylation pattern of hippocampus obtained from PNE and control mice. Principal component analysis (PCA) showing the distribution of the averaged β-values of the CpG sites between the four groups of samples (males and females of control and PNE groups). PC1, principal component 1; PC2, principal component 2.

To identify general methylation patterns, we performed PCA on the overall methylation levels in the hippocampal samples of the four experimental groups revealed a tight clustering according to sex and prenatal nicotine exposure (Fig 2).

### Identification of differentially methylated positions (DMPs) in PNE and control mice in hippocampus

The comparison between hippocampal samples from PNE and control mice revealed a total of 281 DMPs (adjusted P value <0.05 and delta absolute value >0.2) between 276,649 CpG probes (see S1 Table). One hundred twenty-two DMPs were hypomethylated (43.42%), and one hundred fifty-nine displayed a hypermethylated pattern (56.58%) in the hippocampus of PNE mice compared with the controls. The top 20 DMP-associated genes are shown in Table 1.

**Table 1:** List of top 20 DMP-associated genes comparing hippocampal samples of PNE and control mice.

Gene symbol	Probe ID	Delta beta	ENTREZ_GENE ID	Type of gene	Gene description
Trim14	cg40844713	0.5482	74735	Protein coding	Tripartite motif-containing 14
1700011B04Rik	cg31429790	0.5296	100503727	ncRNA	RIKEN cDNA 1700011B04 gene
Adgrb2	cg41465732	0.4443	230775	Protein coding	Adhesion G protein-coupled receptor B2
Smdt1	cg33590375	0.4278	69029	Protein coding	Single-pass membrane protein with aspartate rich tail 1
Lifr	cg33041852	0.4336	16880	Protein coding	LIF receptor alpha
Cpvl	cg43443551	0.4234	71287	Protein coding	Carboxypeptidase, vitellogenic-like
Map3k13	cg33998012	0.4219	71751	Protein coding	Mitogen-activated protein kinase kinase kinase 13
Zg16	cg45127550	0.4197	69036	Protein coding	Zymogen granule protein 16
Vmn1r72	cg44232693	0.4119	252905	Protein coding	Vomeronasal 1 receptor 72
Rab14	cg38276813	0.4103	51552	Protein coding	RAB14, member RAS oncogene family
Erbb3	cg29163869	−0.5408	13867	Protein coding	erb-b2 receptor tyrosine kinase 3
Rnf141	cg44986716	−0.5343	67150	Protein coding	Ring finger protein 141
Maz	cg45127038	−0.5165	17188	Protein coding	MYC-associated zinc finger protein (purine-binding transcription factor)
Rab5c	cg30039301	−0.4945	19345	Protein coding	AB5C, member RAS oncogene family
Tgfbr3	cg42547593	−0.4798	21814	Protein coding	Transforming growth factor, beta receptor III
Donson	cg34491319	−0.4549	60364	Protein coding	Downstream neighbor of SON
Olfr1278	cg38804397	−0.4487	258389	Protein coding	Olfactory receptor family 4 subfamily F member 54
L2hgdh	cg30752476	−0.4413	217666	Protein coding	L-2-hydroxyglutarate dehydrogenase
Dram1	cg28806784	−0.4356	71712	Protein coding	DNA-damage regulated autophagy modulator 1
Postn	cg39769819	−0.4302	50706	Protein coding	Periostin, osteoblast specific factor

An analysis of the genomic distribution of DMPs revealed that the highest proportion were in the body region (Table 2).

**Table 2:** Genomic distribution of significant DMPs in PNE and control mice.

Genomic Regions	Hypomethylated DMPs		Hypermethylated DMPs	
	n	%	n	%
First exon	11	9.0	27	17.0
3’UTR	1	0.8	3	1.9
5’UTR	7	5.7	11	6.9
Body	49	40.2	70	44.0
IGV – open sea	27	22.1	29	18.2
TSS1500	10	8.2	10	6.3

UTR: indicates the untranslated region; Body: indicates the region between ATG and the stop codon; TSS: indicates the transcription start site; TSS1500: refers to 200–1500 bases upstream of the TSS; IGV-open sea: isolated regions without specific designation.

**Fig 3:**
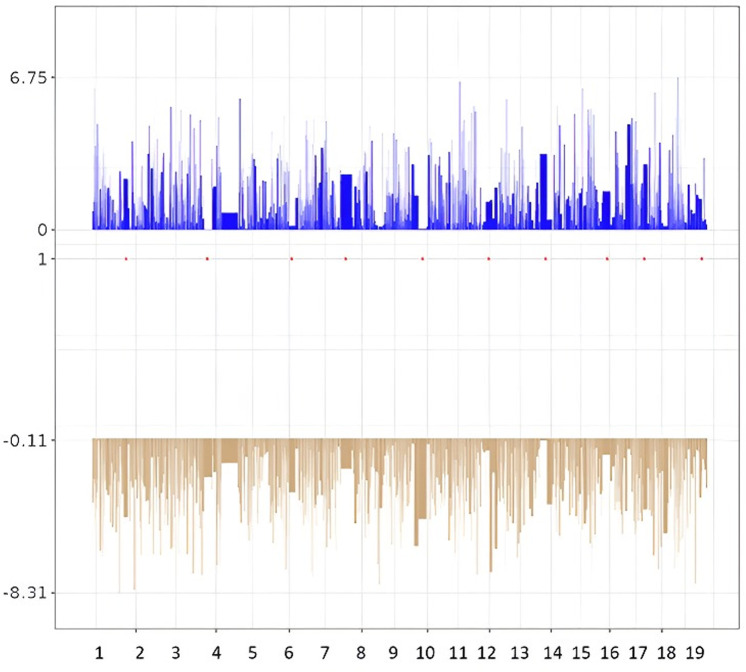
Chromosome distribution of differentially methylated positions between PNE and control mice in hippocampus. The blue and brown lines represent the hypermethylated and hypomethylated DMPs, respectively. Y-axis corresponds to the delta beta-value.

The comparison between PNE and control mice revealed that DMPs are distributed across all chromosomes (Fig 3).

### Functional enrichment analysis of DMPs show association with the nervous system development and ErbB-3-dependent intracellular cascade

**Fig 4:**
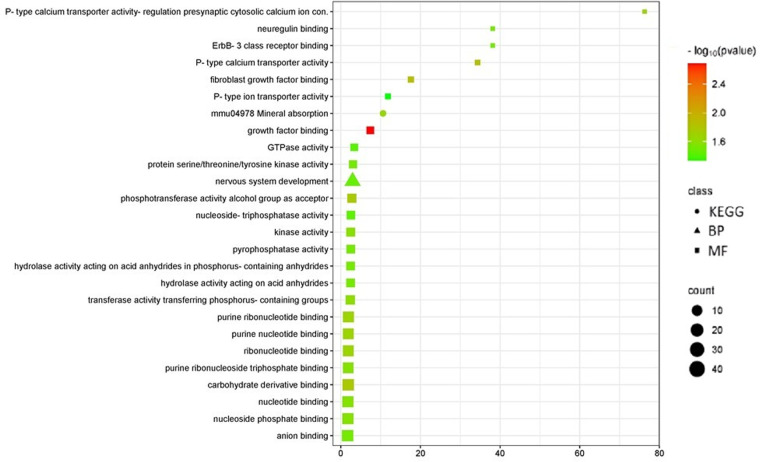
GO and KEGG functional enrichment analysis of DMPs identified in hippocampal samples from PNE and control mice. Percentage of enrichment is on the X-axis; counts number is proportional to symbol size and color code to -log10 of p-value. GO, Gene Ontology; KEGG, Kyoto Encyclopedia of Gene and Genomes; BP, biological process; MF, molecular function.

The GO functional gene enrichment analyses of the DMP-associated genes revealed one biological process (GO:0007399: nervous system development) and 24 molecular functions terms that were significantly enriched (see S2 Table). Abnormal time course of volume increase of neocortex and subcortical areas during childhood of ADHD patients suggesting a delay in neurodevelopment is well documented [15]. In addition, the GO molecular functions terms showed a relevant fold enrichment for GO terms associated to P-type calcium transporter activity involved in the regulation of presynaptic cytosolic calcium ion concentration (GO:1905056), neuregulin binding (GO:0038132) and ErbB-3 class receptor binding (GO:0043125). Interestingly, Neuregulin-ERBB signaling is involved in several neural processes such as myelination, neurotransmission and synaptic plasticity and neuropsychiatric disorders such as schizophrenia, bipolar disorder, and depression. In addition, KEGG enrichment analysis revealed one pathway enriched corresponding to mineral absorption (mmu04978) (Fig 4). Calcium, sodium, and potassium ions are required for the excitability of neuronal cells and chemical neurotransmission.

### Identification of Differentially methylated regions (DMRs) between PNE and control mice in hippocampus

To gain insight into the connections among DMPs and expressed genes in PNE and control animals, we evaluated differentially methylated regions (DMRs). A total of 113 (including 58 hypomethylated and 55 hypermethylated) DMRs were filtered in PNE mice (see S3 Table). Among these DMRs, the top five genes with the highest level of hypomethylation in DHD-mice corresponded to *PPCDC, TMOD3, MYL3, TTC9C* and *CACNA1I*. Similarly, the top five genes with the highest level of hypermethylation in PNE mice corresponded to *PHC3, H2-D1, RPGRIP1I, HAND1* and *MAPT.* We further performed GO and KEGG functional enrichment analyses of these DMRs. The GO functional gene enrichment analyses of the DMR-associated genes revealed one biological process (GO:0035332: positive regulation of hippo signaling; Fold Enrichment: 96) and three molecular functions terms significantly enriched (GO:000378: actin monomer binding; Fold Enrichment: 28) (GO:0019838 growth factor binding; Fold Enrichment: 139) (GO:0008092 cytoskeletal protein binding; Fold Enrichment: 3.1). KEGG analysis did not revealed any enriched pathway.

To validate the relationships among DMR genes and their transcript levels, one hypermethylated (*CORO7*) and two hypomethylated (*ERBB3* and *MYL4*) DMR genes were selected.

**Fig 5:**
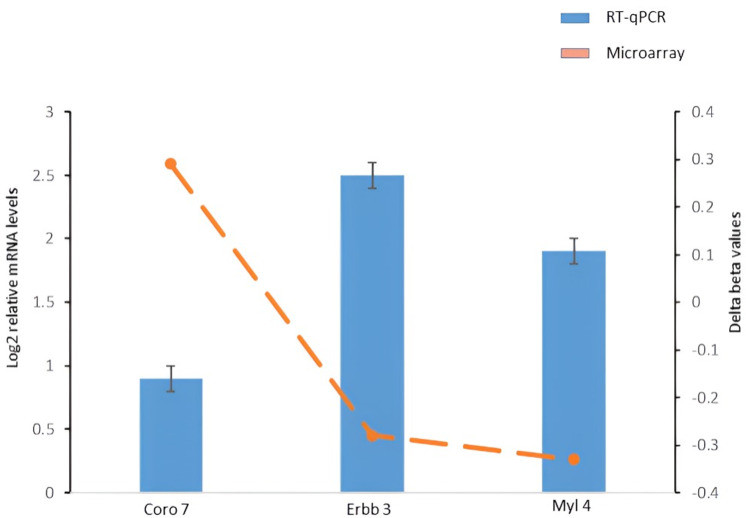
Gene expression analysis for the selected DMR-associated genes *CORO7*, *ERBB3* and *MYL4* in hippocampus of PNE and control mice. The delta of beta-values for *CORO7*, *ERBB3* and *MYL4* obtained from the microarray data (PNE versus control mice) are plotted as blue solid bars. Relative mRNA levels were obtained from levels of transcripts (evaluated by RT-qPCR) in PNE samples normalized to levels detected in control samples are plotted as orange solid circles.

All the primer pairs designed for the three DMR genes chosen for RT-qPCR validation had efficiencies between 94.57 and 98.02%, which is within the ideal range for qPCR analysis. The relative mRNA levels of the selected genes agreed with the microarray results supporting the relationship between DNA methylation status and gene expression level of target genes (Fig 5).

## Discussion

ADHD corresponds to a neurodevelopmental disorder with a high index of heritability (approximately 80%) and several risk genes have recently been identified using GWAS approaches. Among those genes (mainly expressed in excitatory and inhibitory neurons) there are transcription factors expressed in brain tissue and structural components of postsynaptic density previously associated with neurodevelopmental disorders [5]. However, the effects of extrinsic factors during pregnancy and postnatal development on the incidence of this disorder through epigenetic modifications are poorly understood. In this work we analyzed DNA methylation in brain samples from the PNE murine model of ADHD to identify putative epigenetic biomarkers of the disorder to develop further clinical studies in humans. The use of animal models for human neurological disorders or diseases is limited considering the divergence in brain neuroanatomy, timeline of neurodevelopment and cognitive properties. However, the current use of murine models for neurodevelopmental and neurodegenerative diseases such as Parkinson’s disease, Alzheimer’s disease and Amyotrophic Lateral Sclerosis has been useful for describing the molecular pathways underlying these disorders and for the development and testing of new drugs in preclinical studies [26].

By another part, several reports focused on the study of the epigenome in ADHD have used saliva or blood samples from patients or postmortem brain samples [9,12,16]. The present study employed fresh hippocampal tissue dissected from control and PNE mice. Considering that DNA methylation patterns are cell type specific our results are relevant in terms of describing the DNA methylation-associated epigenetic changes that occurs in the hippocampus, one of the subcortical areas significantly reduced in volume in children and adults with ADHD [15].

GO analysis of hippocampal DMPs revealed the nervous system development as a biological process, was significantly enriched in PNE samples which is consistent with the categorization of ADHD as a neurodevelopmental disorder. A total of 281 DMPs, almost 57% of which were hypermethylated, located principally in the body region, were detected. Methylation localized in gene body regions will alter the gene transcription process through the optimization of alternative splicing events or transcription and elongation [27]. Among the 20 top DMP-mapped genes, several loci associated with neural development and physiology such as *ERBB3* (erb-b2 receptor tyrosine kinase 3), *ADGRB2* (adhesion G protein-coupled receptor B2) and *LIFR* (leukemia inhibitory factor receptor) were identified. *ADGRB2* codes for the adhesion-GPCR member B2 receptor (BAI2) and belongs to a subfamily of GPCRs (BAI1-3) highly expressed in the brain and involved in synaptogenesis. BAI-2 suppresses the expression of VEGF and *BAI2 KO* mice exhibit an enhanced adult neurogenesis in the hippocampus associated with the recovery of VEGF-dependent neurogenesis [28]. In addition, LIF signaling is involved in the proliferation and differentiation of human cortical stem cells (or basal radial glia) in excitatory neurons, astrocytes and oligodendrocytes [29]. Recently, it has been reported that LIF promotes the differentiation of these cells into inhibitory interneurons in cortical primary cultures and forebrain organoids [30]. These results are consistent with the etiology of ADHD, which is considered a neurodevelopmental disorder characterized by a decreased volume of the neocortex and subcortical areas such as the hippocampus [15]. In addition, adolescents with ADHD exhibit smaller gray matter volume in several brain regions correlated with lower performance in working memory tests [31]. This impairment in working memory is consistent with our previous reports showing that PNE mice exhibit a significant impairment in hippocampal LTP with a lower density of dendritic spines in CA1 pyramidal neurons [19,32].

Although DMP analyses are frequently reported in epigenomic studies, DMR analyses are biologically more robust, showing a higher correlation with gene expression levels, because of the substantial correlation between contiguous CpGs [33]. In the present study we detected a total of 113 DMR-genes (58 hypomethylated and 55 hypermethylated) between PNE and control mice in hippocampus. Notably only one of these DMR-genes, *SPTBN2* (spectrin beta non-erythrocytic 2) have also been found in children with ADHD in a hypomethylated status by analysis of DNA methylation in blood samples [34]. The *SPTBN2* gene encodes for the beta-III spectrin (protein forming part of cytoskeleton), which is expressed mainly in the cerebellum (affecting glutamate turnover) and several mutations have been associated with Spinocerebellar Ataxia Type 5 (SCA5) in humans [35]. *SPTBN2-KO* mice exhibit ataxia and progressive degeneration of Purkinje cells in the cerebellum. In addition, neurons from the prefrontal cortex (involved in attention) of these mice show abnormal dendritic development and memory impairment as evaluated in object recognition-based tests [36]. In addition, among the genes significantly hypomethylated in our DMR analysis, the *CACNA1i* gene was detected. *CACNA1i* codes the alpha1 subunit of the Ca_v_3.3 (T-type) voltage-dependent calcium channel and is involved in the modulation of the neuronal firing pattern [37]. These calcium currents in CA1 pyramidal neurons can be reduced by the activity of D5 dopamine receptors decreasing the firing rate of these cells [38].

The GO biological process terms and KEGG enrichment analysis of DMRs included genes associated with positive regulation of the Hippo pathway as the main biological process distinctive between control and PNE mice. The Hippo signaling pathway is involved in several cellular processes such as cell proliferation, differentiation and migration during development and organ regeneration [39]. Interestingly, the downstream YAP1/TAZ transcriptional coactivators of the Hippo pathway are expressed in adult hippocampal neural stem cells (NSCs) and stimulate their activation [40]. In addition, the WW and C2 domain-containing protein 1 (WWC1) have been described as upstream regulatory components of Hippo intracellular cascade [39]. Cell surface proteomics analysis of hippocampal tissue suggested that WWC1 facilitates the anchoring of AMPA receptors in the postsynaptic density enhancing learning and memory in mice [41]. In addition, it has been reported that *WWC1/2 KO* mice exhibit lower spine density in the hippocampus and impaired learning and memory [42]. Consistently, the *CORO7* gene (encoding coronin 7, a scaffold protein involved in the Hippo intracellular cascade) which in our analysis exhibited a hypermethylated status (PNE compared with control samples) was correlated with lower levels of transcripts as expected (see Fig 4). Our previous reports suggest a delay in spine maturation in CA1 hippocampal neurons of PNE mice compared with controls [19]. In this context, to evaluate the modulation of spinogenesis and spine maturation in PNE animals by the Hippo pathway as an epigenetic target of nicotine exposure during pregnancy became relevant. Moreover, among the molecular functions emerged from enrichment GO analysis with highest rich factor, the P-type calcium channels, the neuregulin binding and the ErbB3 membrane receptor binding were found. These proteins are relevant in the context of synaptic plasticity processes such as hippocampal LTP. The P/Q-type calcium channels (Ca_v_2.1) are expressed mainly in presynaptic terminals involved in the Ca^2+^-triggered fusion of vesicles containing neurotransmitters during the chemical synaptic transmission in the central and peripheral nervous systems [43]. The conditional *CAV2.1 KO* mice exhibit impaired synaptic transmission and deficits in learning and memory processes [44]. In addition, several reports support the regulation of hippocampal neurogenesis and synaptic plasticity by growth factors such as BDNF and neuregulins [45,46]. ErbB3 is a membrane receptor expressed in the hippocampus and its activation by neuregulin-1 is involved in neurogenesis in the ventral dentate gyrus [47]. Interestingly, the *NGR3* gene (encoding for neuregulin 3 ligand of the ErbB3 receptor) has been reported as a DMR in the EWAS of postmortem brain samples from individuals with ADHD [16].

The DNA methylation data included in this report used as input DNA samples from whole hippocampal homogenates. Mouse hippocampus comprises glutamatergic neurons, GABAergic interneurons, astrocytes, oligodendrocytes, vessels-associated cells, and other cell types. Considering that the estimated proportion of neuronal cells corresponds to approximately 50% [48], the contribution of non-neuronal cells to the CpG-associated signals in our analysis must be considered. Further single-cell epigenome and transcriptome analyses will be needed to determine the cell-specific epigenetic modifications.

In addition, the DNA methylation analysis of other brain areas such as the prefrontal cortex (involved in attention) in PNE mice to determine whether the epigenetic modifications found in the hippocampus are specific is also needed. Furthermore our previous studies revealed that PNE mice exhibit motor hyperactivity, inattention and impaired hippocampal LTP evaluated in ex-vivo experiments [19]. These defects were not observed in PNE animals treated with a single oral administration of methylphenidate, which is used as a pharmacotherapy for ADHD in children [49]. Therefore, further analysis of epigenetic modifications in PNE mice treated with methylphenidate will be useful for determining whether the recovery of abnormal behavior and hippocampal synaptic plasticity involves changes in the epigenetic status of the same DMPs and DMRs described in this report.

## Supporting information

S1 Fig Behavioral characterization of prenatal nicotine exposure (PNE) and control mice. (A) Top, Video tracking of spontaneous locomotor activity. Bottom. Quantification of the average total distance traveled. Significant differences were observed between PNE animals and control mice (PNE: 78.37 ± 2.01 m; n = 6; Control = 51.57 ± 3.33 m; n = 6), t = 6,883 df = 10, p < 0,0001. (B) Top, Diagram of the object-based attention test experimental protocol and video tracking of the retention stage. Bottom, PNE mice showed a decreased recognition index percentage compared to control mice (PNE: 24.00 ± 1.03%, n = 6; Control: 53.17 ± 4.09%, n = 6), t = 6,921 df = 10, p < 0,0001. The detailed descriptions of the behavioral tests are described in Contreras et al. (2022).
(TIF)

S1 Table List of differentially methylated positions (DMPs) identified in hippocampal samples from PNE and control mice. (XLSX)

S2 Table Functional gene enrichment analysis of DMP-associated genes identified in hippocampal samples from PNE and control mice. (XLSX)

S3 Table List of differentially methylated region (DMR)-associated genes identified in hippocampal samples from PNE and control mice. (XLSX)
