# Supplementary figures and images for: Genome-wide DNA methylation analysis of hippocampal tissue in a murine model of attention deficit-hyperactivity disorder
Source: PLoS One. 2025 Jun 4;20(6):e0323756. doi: 10.1371/journal.pone.0323756 (PMC12136424; doi:10.1371/journal.pone.0323756)

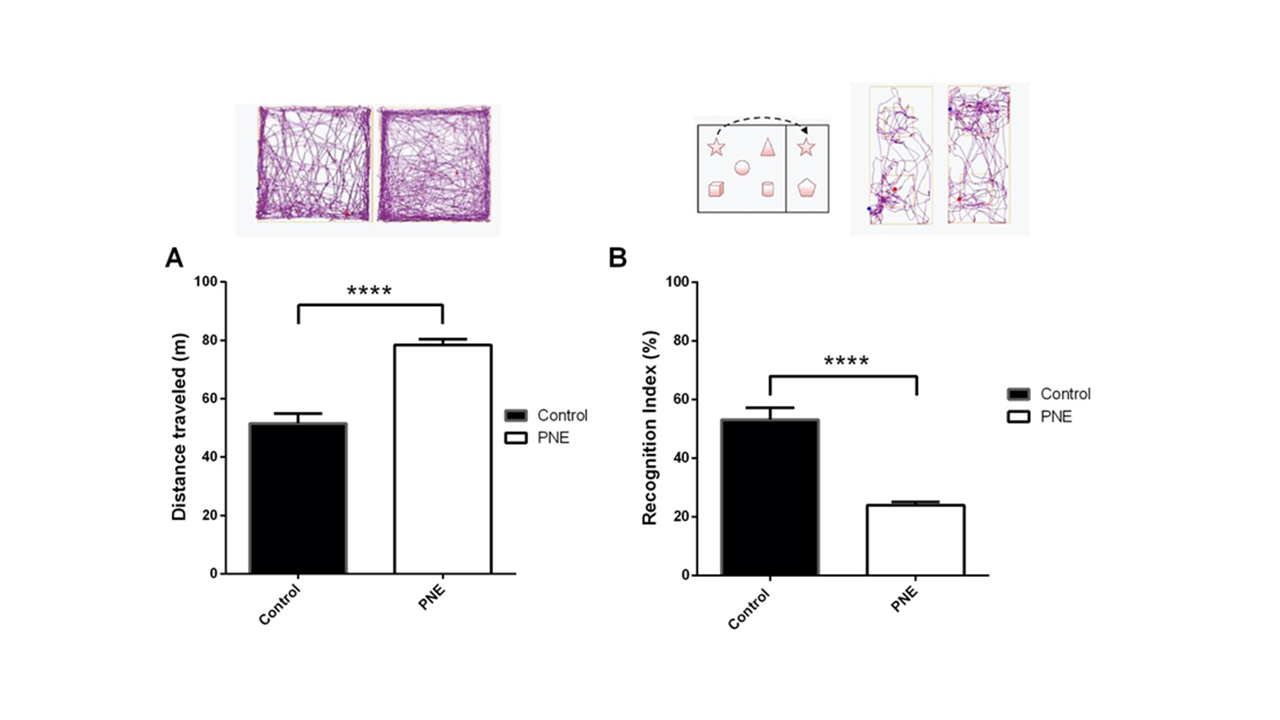

Supplement: S1 Fig — (A) Top, Video tracking of spontaneous locomotor activity. Bottom. Quantification of the average total distance traveled. Significant differences were observed between PNE animals and control mice (PNE: 78.37 ± 2.01 m; n = 6; Control = 51.57 ± 3.33 m; n = 6), t = 6,883 df = 10, p < 0,0001. (B) Top, Diagram of the object-based attention test experimental protocol and video tracking of the retention stage. Bottom, PNE mice showed a decreased recognition index percentage compared to control mice (PNE: 24.00 ± 1.03%, n = 6; Control: 53.17 ± 4.09%, n = 6), t = 6,921 df = 10, p < 0,0001. The detailed descriptions of the behavioral tests are described in Contreras et al. (2022). (TIF) [file pone.0323756.s001.tif]
